# Anisotropy Engineering of ZnO Nanoporous Frameworks: A Lattice Dynamics Simulation

**DOI:** 10.3390/nano12183239

**Published:** 2022-09-18

**Authors:** Na Sa, Sue-Sin Chong, Hui-Qiong Wang, Jin-Cheng Zheng

**Affiliations:** 1 Fujian Provincial Key Laboratory of Semiconductors and Applications, Collaborative Innovation Center for Optoelectronic Semiconductors and Efficient Devices, Department of Physics, Xiamen University, Xiamen 361005, China; 2 Department of Physics, Xiamen University Malaysia, Sepang 43900, Malaysia; 3 Department of New Energy Science and Engineering, Xiamen University Malaysia, Sepang 43900, Malaysia

**Keywords:** zinc oxide, nanoporous framework, mechanical properties, anisotropy

## Abstract

The anisotropy engineering of nanoporous zinc oxide (ZnO) frameworks has been performed by lattice dynamics simulation. A series of zinc oxide (ZnO) nanoporous framework structures was designed by creating nanopores with different sizes and shapes. We examined the size effects of varying several features of the nanoporous framework (namely, the removal of layers of atoms, surface-area-to-volume ratio, coordination number, porosity, and density) on its mechanical properties (including bulk modulus, Young’s modulus, elastic constant, and Poisson ratio) with both lattice dynamics simulations. We also found that the anisotropy of nanoporous framework can be drastically tuned by changing the shape of nanopores. The maximum anisotropy (defined by Y_max_/Y_min_) of the Young’s modulus value increases from 1.2 for bulk ZnO to 2.5 for hexagon-prism-shaped ZnO nanoporous framework structures, with a density of 2.72 g/cm^3^, and, even more remarkably, to 89.8 for a diamond-prism-shape at a density of 1.72 g/cm^3^. Our findings suggest a new route for desirable anisotropy and mechanical property engineering with nanoporous frameworks by editing the shapes of the nanopores for the desired anisotropy.

## 1. Introduction

Zinc oxide is a chemically stable and environmentally friendly multifunctional material [1,2,3,4] with excellent physical and chemical properties such as direct wide band gap (3.37 eV), high exciton binding energy (60 meV), high mechanical stability, and good light stability. It is also easily etched in all acids and alkalis, which provides opportunities for manufacturing small-sized devices [1]. ZnO nanostructures [5,6,7] with various morphologies, such as nanocombs [8,9], nanorings [10,11,12], nanospirals [13], nanosprings [14], nanoribbons [15,16], nanosheets [17,18,19,20], nanowires [21,22,23,24,25], and nanocages [26,27], have been extensively studied. In our previous computational work, the structural and electronic properties of ZnO polytypes [28] and ZnO nanostructures including nanowires [29,30], nanotubes [29,31], and nanofilms [32,33] were investigated.

The interfaces between ZnO and other materials are also important topics. [34,35,36,37,38] The structure and electronic properties of interfaces between hexagonal ZnO and cubic oxides have been investigated in detail by a combination of transmission electron microscopy (TEM), synchrotron radiation-based characterization techniques, and density functional theory [39,40]. The surface morphologies and properties of ZnO films can be tuned by the design of the interfacial layer [41]. The surface effects on the mechanical properties of ZnO nanostructures have been investigated [42,43]. Ab initio calculations have also applied to study the structural and electronic properties of ZnO surfaces [44,45,46].

Nanoporous materials have the characteristics of both nanostructures and surface/interface, in some respects. The tuning of the shape of nanostructure or pores and interface is related to the modification to the symmetry of the materials. It was recently reported that the symmetry and asymmetry of an electronic structure can be important for its thermoelectric transport properties [47]. It would be interesting to see how the symmetry of nanopores affect the mechanical properties of nanoporous materials. There are reports on porous structures made of ZnO [17,19,20,48], including nanosheets [17,19,20], hierarchically porous ZnO architectures [48], etc. The key to obtaining an overall high-performance nanoporous material depends on its porous structure [48,49,50,51,52,53,54]. Inspired by cellular microstructures in biomorphic materials [55], we designed a series of zinc oxide (ZnO) nanoporous framework (NPF) structures by creating nanopores with different shapes and sizes in periodic wurtzite ZnO supercells, and then we performed lattice dynamics (LD) simulation to investigate the mechanical properties of the designed NPF structures in terms of several structural parameters.

The anisotropic properties, namely, the strong orientation or direction-dependent properties, are desirable for special devices. The anisotropic strain effects on the photoluminescence of (Zn,Mg)O/ZnO quantum wells have been reported [56]. The anisotropic mechanical properties have also been reported for two-dimensional (2D) materials [57,58]. However, the anisotropic features of ZnO nanostructures, especially three-dimensional (3D) nanoporous materials, are studied less. Therefore, we further analyzed the anisotropy of 3D NPF-ZnO structures with different porous structures, and then we tuned the properties though an anisotropy engineering approach.

## 2. Computational Methods

The LD simulations were performed using a general utility lattice program (GULP) [59] with a partially charged rigid ion model (PCRIM) for ZnO interatomic pair potentials [60]. It has been shown [60] that the structural parameters and mechanical properties of ZnO with different polytypes obtained from this PCRIM model are in good agreement with ab initio calculations and experimental results. Our LD simulations reproduced the results for bulk polytypes well [60], and then we applied the same potentials to the simulation of the NPF structures. The anisotropy of ZnO, defined by Y_max_/Y_min_, was obtained using an open-source online application for the analysis of elastic tensors (the ELATE program) [61].

## 3. Results

### 3.1. Models of Nanoporous Framework Structures

**Figure 1:**
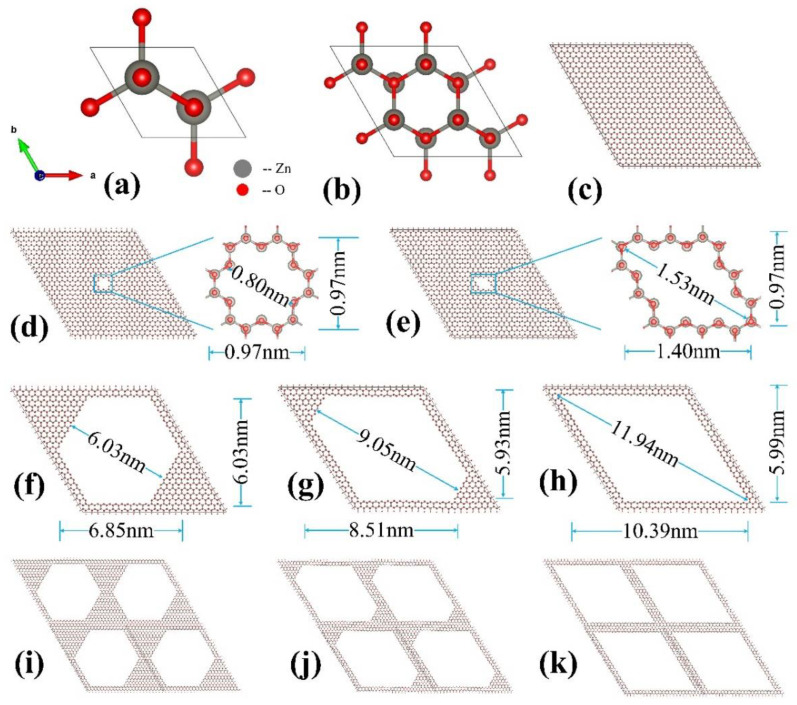
(shown in color online) Schematic illustration of the creation of nanopores in ZnO supercells to form nanoporous framework (NPF) structures. (**a**) Bulk ZnO unit cell. (**b**) Small ZnO supercell (with 2 × 2 × 1-unit cells). (**c**) Typical larger ZnO supercell (with 24 × 24 × 1-unit cells). (**d**) Optimized NPF structure with small hexagon-prism-shaped nanopores in 24 × 24 × 1 supercell, namely, 24 × 24 × 1H. (**e**) Optimized NPF structure with small diamond-prism-shaped nanopores in 24 × 24 × 1 supercell, labeled as 24 × 24 × 1D. (**f**) Optimized NPF structure, 24 × 24 × 1H, with larger nanopores. (**g**) Optimized NPF structure with larger irregular nanopores (transition between H-ZnO and D-ZnO, denoted by T-ZnO) in a 24 × 24 × 1 supercell. (**h**) Optimized NPF structure, 24 × 24 × 1D, with larger nanopores. (**i**–**k**) 2 × 2 (in ab plane of supercell) illusions of (**f**–**h**), respectively. The zinc atom is labeled with a large gray sphere and the oxygen atom is shown with a small red ball.

The ground state of a ZnO crystal has a wurtzite (WZ) structure (space group P63mc) with two zinc and two oxygen atoms per unit cell, as shown in Figure 1a. The lattice constants and internal parameters obtained from calculations are as follows: a = b = 3.238 Å, and u = 0.3814, respectively, which is in good agreement with the PRCIM calculation results of Wang et al. [60]. A series of zinc oxide (ZnO) NPF structures were created by making nanopores with different shapes and sizes in periodic WZ-ZnO supercells, as shown in Figure 1. We used different supercell structures (i.e., 8 × 8 × 1-, 12 × 12 × 1-, 16 × 16 × 1-, 20 × 20 × 1-, and 24 × 24 × 1-unit cells) to construct three-dimensional zinc oxide (ZnO) NPF structures. Because symmetry is important for the properties of functional materials [47], we also considered two typical shapes of NPF structures with different symmetries of pores, i.e., diamond-prism-shaped (D-ZnO) and hexagon-prism-shaped (H-ZnO) porous nanomaterials. The different NPF structures constructed remained of the same composition ratios as that of a bulk structure, namely, Zn:O = 1:1. The labels of the NPF structures are denoted as n × n × 1D for D-ZnO and n × n × 1H for H-ZnO, where n is the number of unit cells. The supercells in the z-direction were repeated with 1 unit cell, resulting in a 3D prism shape. The initial NPF-ZnO structures were then relaxed to obtain geometry-optimized stable structures.

### 3.2. Bulk Moduli of Nanoporous Framework Structures

**Figure 2:**
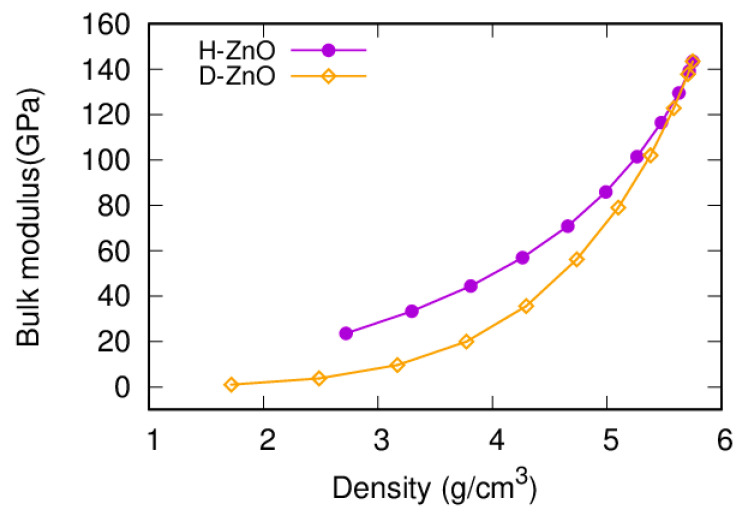
(Color online). The bulk moduli of the NPF-ZnO structures (H-ZnO vs D-ZnO) in terms of the size and shape of the nanopores as a function of the density (24 × 24 × 1 supercells are used for comparison).

The bulk modulus is an important mechanical property and is a useful indicator of the hardness of a material [62,63,64,65,66]. The bulk modulus of the bulk ZnO obtained in this work is 143.5 GPa, which is in good agreement with previous experimental values (141 GPa [67] and 142.6 GPa [68]), as well as the previous PCRIM (143.5 GPa) [60], ReaxFF (144 GPa) [69], and ab initio calculations (127 GPa [29], 133.7 GPa [70], 149 GPa [71], and 161 GPa [72]). It was interesting to find that the bulk modulus is sensitive to the size and shape of the nanopores. The trends of the bulk moduli for the NPF-ZnO structures with differently sized and shaped nanopores shared several common features (Figure 2). Firstly, the bulk moduli of the NPF-ZnO structures decreased with the increasing size of the pores (decreasing density). Secondly, the decreasing trends of the bulk moduli were quite similar for the NPF-ZnO structures of different shapes, namely, the hexagon-prism shape and diamond-prism shape (n × n × 1H and n × n × 1D supercells). At the same density, the bulk moduli of the D-ZnO structure (n × n × 1D) were much smaller than those of the H-ZnO (n × n × 1H) NPF structures. which means that D-ZnO is softer than H-ZnO.

### 3.3. Young’s Moduli and Elastic Constants of the Nanoporous Framework Structures

**Figure 3:**
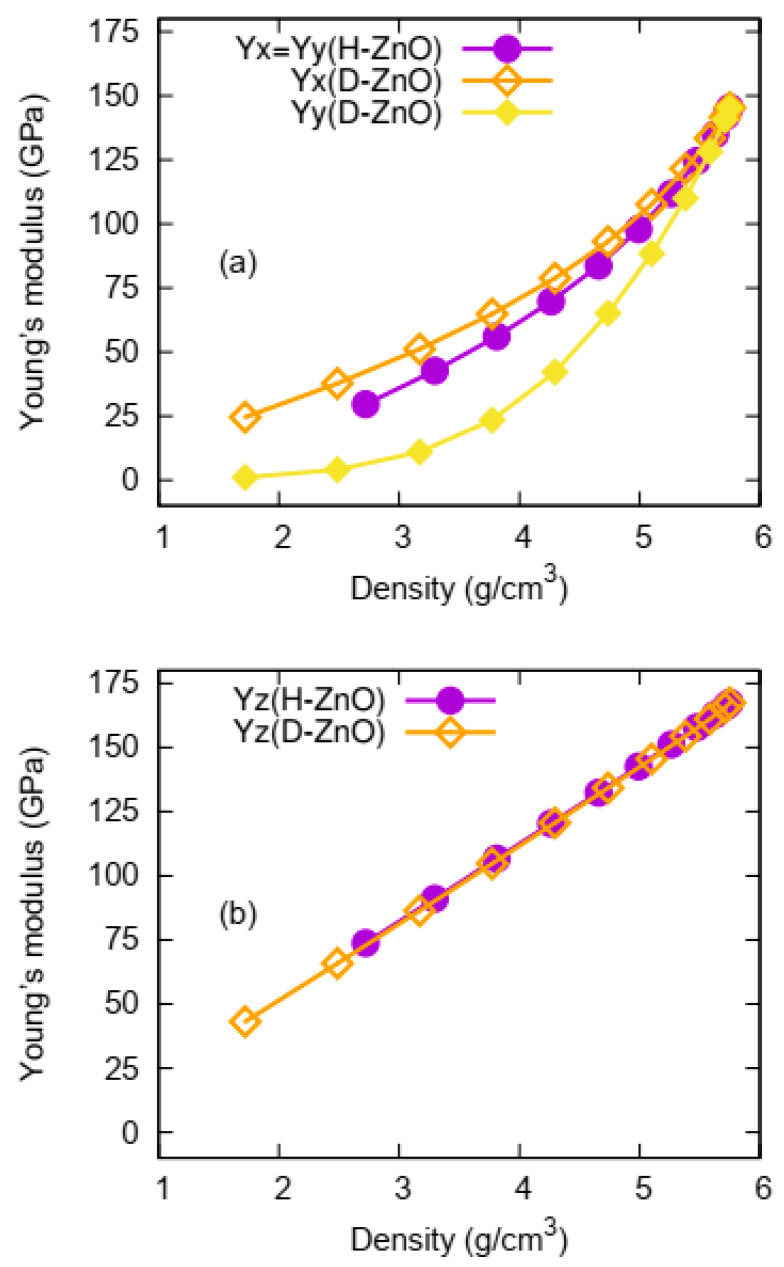
(shown in color online) The Young’s moduli of the NPF-ZnO structures in terms of the sizes and shapes of the nanopores as a function of the density. (**a**) H-ZnO. (**b**) D-ZnO.

The calculated Young’s moduli as a function of the sizes and shapes of the NPF-ZnO structures are shown in Figure 3. Similar trends can be found for the Young’s modulus as a function of the density with that of the bulk modulus. However, different from the bulk modulus, which is a volumetric property, the Young’s modulus is direction-dependent and very sensitive to shapes and symmetries of nanopores. As shown in Figure 3a, the Young’s modulus in the x direction (Yx) for D-ZnO is higher than the Yx of H-ZnO, which is different from the aforementioned case of the bulk modulus. On the other hand, the Young’s modulus in the y direction (Yy) for D-ZnO is smaller than the Yy of H-ZnO, which is similar to the case of the bulk modulus. Different from the cases of both the Yx and Yy, the Young’s moduli in the z direction (Yz) are almost the same for both D-ZnO and H-ZnO.

**Figure 4:**
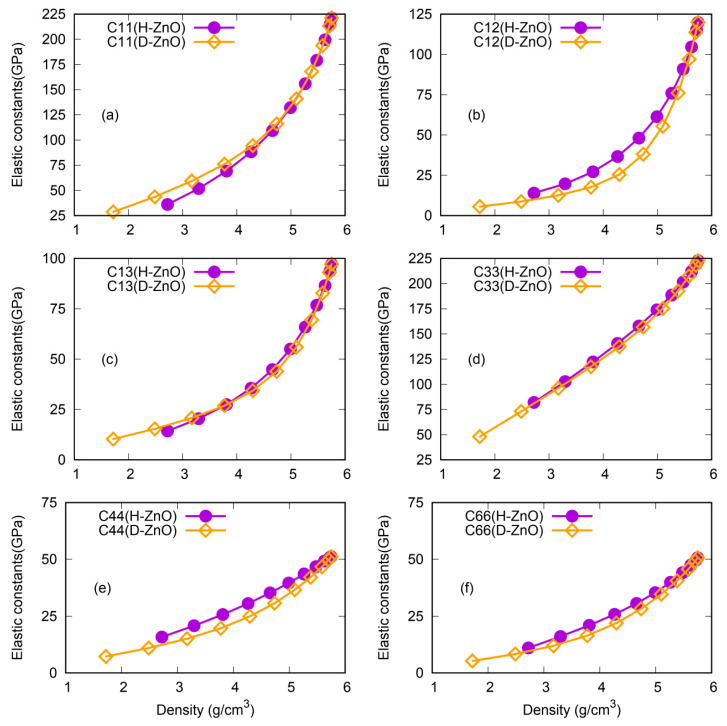
(shown in color online) The comparison of the elastic constants (Cij) of the NPF structures for H-ZnO and D-ZnO as functions of the density. (**a**) C11, (**b**) C12, (**c**) C13, (**d**) C33, (**e**) C44, and (**f**) C66.

The more detailed, but complicated, direction-dependent elastic properties can be observed from the elastic constants of H-ZnO and D-ZnO, as shown in Figure 4. In terms of direction-dependent elastic properties, their responses to the shapes of the nanopores are more sensitive and complicated. For NPF-ZnO with a high mass density (small size of pores), the elastic constants of H-ZnO and D-ZnO are quite similar. However, the elastic constants diverge as the pore size increases (density decreases). For example, the C11 (Figure 4a) of H-ZnO is very close to that of D-ZnO for a density of larger than approximately 4.5 g/cm^3^, but the obvious softness of the C11 of H-ZnO can be observed for a density of smaller than 4.5 g/cm^3^, indicating that the C11 of NPF-ZnO with hexagonal prism pores is more sensitive to density changes (pore sizes) than that of NPF-ZnO with diamond-shaped prism pores. For the C12, as shown in Figure 4b, it is different from the C11. The C12 values of H-ZnO are higher than those of D-ZnO in the calculated range. While for the C13 and C33, the values for H-ZnO and D-ZnO are quite close to each other. The cases of C44 and C66 are similar to the case of C12, i.e., the value of H-ZnO is higher than that of D-ZnO at the same density.

### 3.4. Anisotropy of Nanoporous Framework Structures

**Figure 5:**
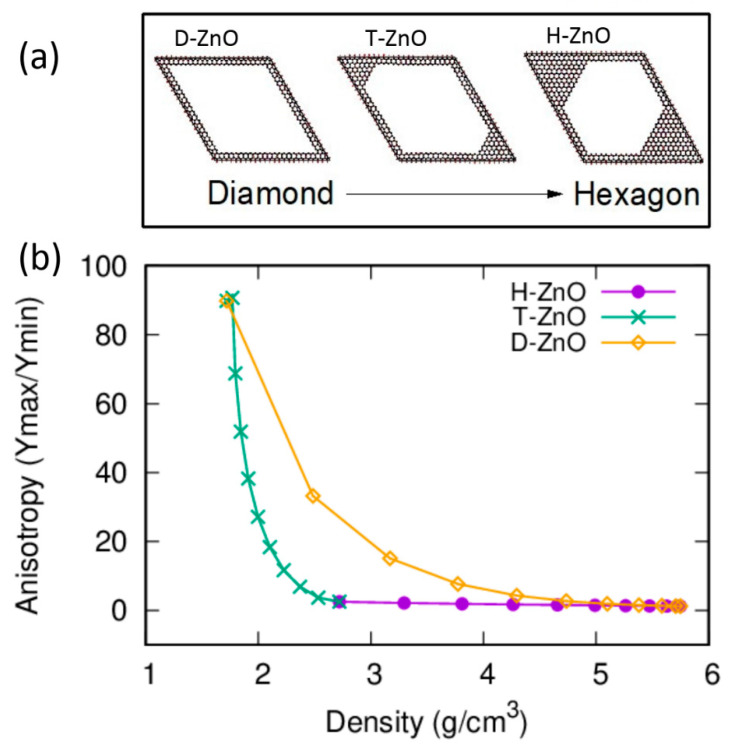
(shown in color online) The comparison of the anisotropy of the mechanical properties between the NPF structures D-ZnO and H-ZnO, as well as that of the transition structure between them. (**a**) The illustration structure changes from 24 × 24 × 1D (labeled as D-ZnO) to a typical transition structure (labelled as T-ZnO) between 24 × 24 × 1D and 24 × 24 × 1H, and then to the end structure, 24 × 24 × 1H (H-ZnO). (**b**) The maximal anisotropy (Ymax/Ymin) of the Young’s modulus for D-ZnO, T-ZnO, and H-ZnO.

The ability to modify anisotropy is desirable for tuning a material’s properties [56,57,58,73]. Therefore, another interesting finding of this work is that the anisotropy of NPF-ZnO can be tuned in a wide range of ways by designing differently shaped nanopores. The anisotropic mechanical properties are typical in the Young’s modulus (Y), which are strongly direction-dependent and sensitive to the shape of the nanopores. In bulk ZnO, due to the hexagonal symmetry of its bulk crystal structure, the Young’s moduli in the x and y directions (Yx and Yy) are the same (isotropic), but the Young’s modulus in the z direction (Yz) is different, and is much higher than those of Yx and Yy. The anisotropy of the Young’s modulus in NPF-ZnO, with its hexagon-prism shape, is quite similar to the bulk ZnO, i.e., with almost identical Young’s moduli in the x and y directions (Yx = Yy), but different from the Yz. The anisotropy of the Young’s modulus of NPF-ZnO, with its diamond-prism shape, is further enhanced by the obvious splitting of Yx and Yy as functions of the density. By defining the ratio of maximum value to minimum value as the maximum value of the anisotropy [61], we can calculate the anisotropy of the Young’s modulus of NPF-ZnO with different pore shapes. The anisotropy of the Young’s modulus of the NPF-ZnO structures is, therefore, presented in Figure 5 for D-ZnO and H-ZnO. The variation of the anisotropy with the transition from D-ZnO to H-ZnO is also shown in Figure 5 for comparison. The anisotropic value calculated by Ymax/Ymin is 1.2 for the bulk ZnO and the supercells without nanopores. For H-ZnO NPF structures, the maximum anisotropy slowly increases to approximately 2.5, with a very low density (2.72 g/cm^3^) within the 24 × 24 × 1H supercell, while the Yx and Yy remain isotropic (ratio = 1). The anisotropy of D-ZnO in all directions varies greatly with the decrease in density, with the maximum value (Ymax/Ymin) reaching as high as 89.8 at a density of 1.72 g/cm^3^. To further explore the anisotropy changes caused by different structures, we designed a series of transition structures (T-ZnO) between H-ZnO and D-ZnO, as shown in Figure 5. As expected, the anisotropic values ranging between those of H-ZnO and D-ZnO were obtained for T-ZnO.

## 4. Discussions

### Universal Features of the Characterization of the Nanoporous Framework

In the previous sections, we presented the properties of an NPF−ZnO structure as a function of the density. However, there are several possible ways to characterize the crystalline features of an NPF−ZnO structure, namely, by defining the (i) layers of atoms removed, (ii) bonding ratio (CN3/CN4 ratio), (iii) surface-area-to-volume ratio (SA/V ratio), (iv) porosity, or (v) density. How to describe the unique structural features of NPF structures is an interesting question. We will compare the above different means (from more atomic details to a more statistical respect) to seek the more universal features of the characterization of NPF−ZnO structures in terms of their mechanical properties.

The layers of atoms removed refers to the number of ZnO atomic layers removed, starting from the center of the supercell, to make a pore. The CN3/CN4 ratio is the ratio of the three-bonded zinc atoms (the atoms in the surface region with three coordination numbers, denoted as CN3) to the four-bonded zinc atoms (the atoms in the bulk-like environment with four coordination numbers, denoted as CN4) contained in the supercell’s structure. The surface-area-to-volume ratio (denoted as “SA/V” in this work) is the amount of surface area per unit of volume. The formula can be defined as SA/(Vt-Vv), where SA is the total surface area of the pores, Vt is the volume of the supercell, and Vv is the volume of the pore (void). The porosity is defined by the ratio denoted as Vv/Vt.

**Figure 6:**
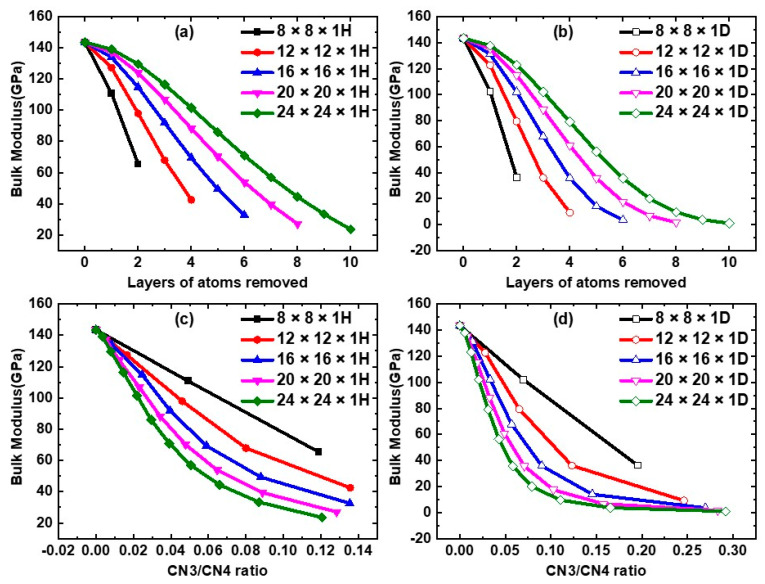

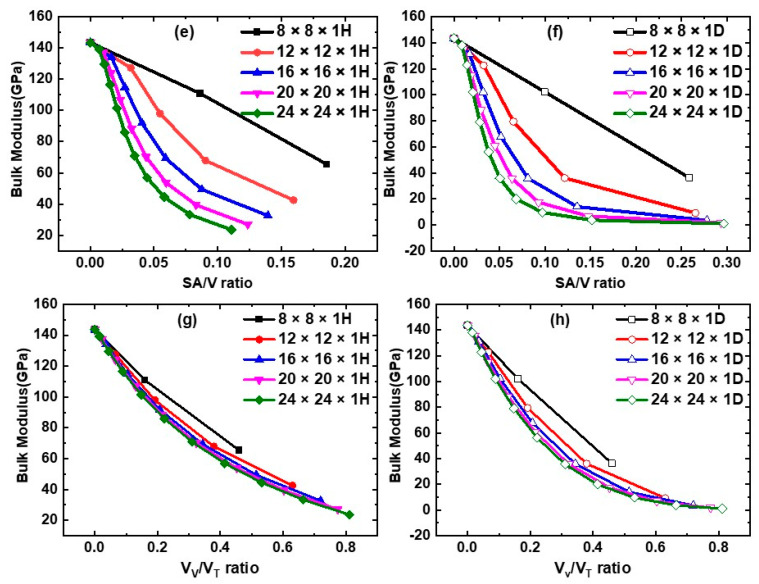
(shown in color online) The variation of the bulk modulus of the NPF−ZnO structure in the n × n × 1D and n × n × 1H unit cells in terms of the different characterizations. (**a**,**b**) The removed layers of atoms. (**c**,**d**) The bonding ratio (CN3/CN4 ratio). (**e**,**f**) The surface-area-to-volume ratio (SA/V ratio). (**g**,**h**) The porosities, respectively. Regarding the structural models, (**a**,**c**,**e**,**g**) are the hexagon-prism-shaped NPF−ZnO (H−ZnO) structures, and (**b**,**d**,**f**,**h**) are the diamond-prism-shaped NPF-ZnO (D−ZnO) structures.

The bulk moduli of the NPF ZnO structures with different characterizations are shown in Figure 6. It is interesting to see that the bulk moduli of the NPF ZnO structures diverge for the different unit cells of n × n × 1D and nn × 1H, being characterized by (i) the layers of atoms removed (Figure 6a,b), (ii) the bonding ratio (CN3/CN4 ratio) (Figure 6c,d), and (iii) the surface-area-to-volume ratio (SA/V ratio) (Figure 6e,f). If the bulk moduli of the NPF ZnO structures are compared in terms of porosity (Figure 6g,h), then the divergence is significantly reduced. The Young’s moduli and elastic constants also follow the same trend.

**Figure 7:**
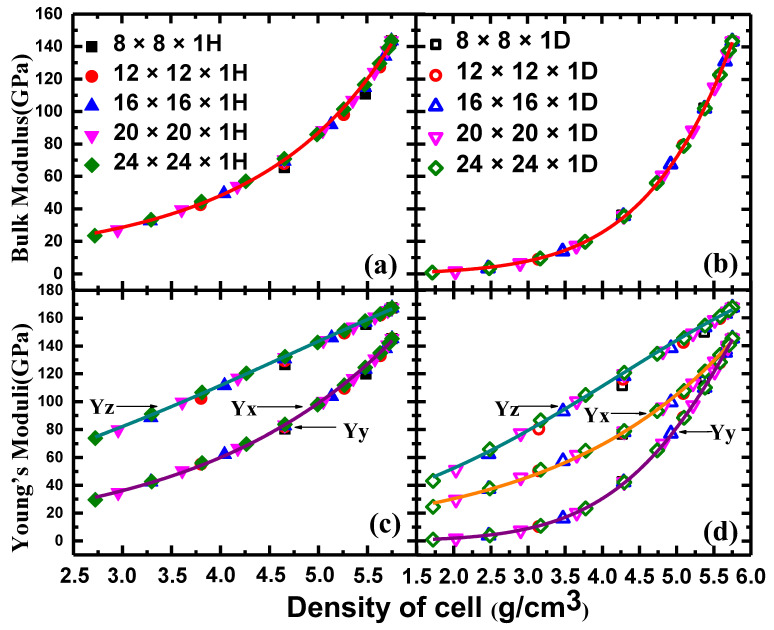
The variation of the bulk modulus and Young’s modulus as functions of density in the n × n × 1D and n × n × 1H unit cells. (**a**,**c**) Hexagon-prism-shaped NPF-ZnO (H-ZnO) structures. (**b**,**d**) Diamond-prism-shaped NPF-ZnO (D-ZnO) structures.

Surprisingly, in the case of the NPF ZnO structure being described by the density of the cells, the mechanical properties are well converged into a unified curve for the different sizes of the unit cells (n × n × 1D or n × n × 1H). The unified pictures, taking the bulk modulus and Young’s modulus as examples, are shown in Figure 7. The bulk modulus of the NPF ZnO structure, obtained from a differently sized supercell, including 8 × 8 × 1H, 12 × 12 × 1H, 16 × 16 × 1H, 20 × 20 × 1H, and 24 × 24 × 1H (with different symbols, as shown in Figure 7a), can be well-fitted in a converged curve (shown by a line). For D−ZnO, ranging from 8 × 8 × 1D to 24 × 24 × 1D supercells, a similar finding can be obtained. For the Young’s moduli of H−ZnO and D−ZnO, the differently sized supercells with different pore sizes can be well-converged into two unified curves (Yz, Yx, or Yy) and three unified curves (if the density is used as a variance, respectively) (Figure 7c,d). Therefore, we have clearly demonstrated that the density of a cell can be a more universal feature for characterizing NPF−ZnO structures in terms of their mechanical properties.

## 5. Conclusions

In conclusion, we systematically investigated the influence of the sizes and shapes of nanopores on the anistropy and mechanical properties of highly ordered NPF−ZnO structures. We discovered that sizes and shapes of nanopores are very sensitive knobs for the mechanical properties of NPF−ZnO structures. The bulk modulus, Young’s modulus, and elastic constants decrease monotonically with decreasing density in both H−ZnO and D-ZnO. The responses of the direction-dependent elastic properties to the shapes of the nanopores are more sensitive and complicated. The anisotropy of an NPF−ZnO structure can be tuned by shaping its nanopores. We also demonstrated that the density of a cell can be a more universal feature for characterizing NPF−ZnO structures in terms of their mechanical properties. Our results shed light on the simulation of a nanoporous framework with desirable anisotropy and mechanical properties.

## Data Availability

The data that support the findings of this study are available from the corresponding author upon reasonable request.
